# The Effect of Hand Dominance on Functional Outcome Following Single Row Rotator Cuff Repair

**DOI:** 10.2174/1874325001611010562

**Published:** 2017-07-25

**Authors:** Michael A. Kelly, Ciarán K. Mc Donald, Aidan Boland, Patrick J Groarke, Ken Kaar

**Affiliations:** 1 Department of Trauma and Orthopedics, UCH Galway, Galway, Ireland; 2 Department of Biomedical Statistics, UCD, Dublin, Ireland

## Abstract

**Introduction:**

Rotator cuff tears are a common cause of shoulder disability and pain. Excellent outcomes can be obtained with surgical treatment although this outcome is affected by several factors. We sought to investigate the effect of hand dominance on subjective functional outcome post rotator cuff repair.

**Methods:**

All patients who had rotator cuff repair over a calendar year were identified and followed up at 3 years post operatively. Patients were consented for inclusion in the study and demographic data, hand dominance and functional outcome data was collected. L’insalata shoulder questionnaire was used for outcome data collection. SPSS version 22 was used for statistical analysis where appropriate.

**Results:**

144 patients were included in this study. Mean age was 63 +/- 10.1 years in the dominant side group and 62 +/- 8.6 years in the non-dominant group. 92 patients had dominant side surgery and 52 had non-dominant side surgery. There was a statistically significant correlation between dominant hand and operated side (P=0.005). The mean overall outcome score was marginally higher in the dominant surgery group with a mean of 89.8 +/- 14.2 compared with a mean of 87.4 +/- 17.5 in the non-dominant group. Multi-variate linear regression analysis revealed this difference to be non-significant (p = 0.4).

**Conclusion:**

No difference was found in the functional outcome of rotator cuff repair between dominant and non-dominant side surgery. This information will help in counselling patients who are concerned about the potential impact of rotator cuff repair on the function of their dominant hand.

## 1 INTRODUCTION

Rotator cuff tears are a common pathology associated with degenerative changes in the shoulder joint. They cause significant disability, pain and poor health status [1, 2] and their prevalence is increasing within an aging population [3, 4].

Many studies have documented excellent outcomes following rotator cuff repair surgery [5, 6], however complications can occur [7]. It is a costly procedure [8] and an increased role for non-operative treatment has been proposed [9].

The risk of rotator cuff tear is much higher on the dominant side [10]. Established factors which affect the outcome following rotator cuff repair include tear size, age and time from tear to surgery [11-13].

During the recovery period, surgery patients are unable to use their arm freely with patients often immobilised in a sling for up to 8 weeks [14]. It could be assumed that patients who have rotator cuff repair surgery on their dominant side will be more limited in terms of ability to perform their activities of daily living during the immobilisation and recovery period. We hypothesised that due to differences in expectations and the physical demands placed upon patients’ dominant hand following surgery, functional outcome differences may exist between dominant and non-dominant hand patients beyond the initial rehabilitation period.

Thus, we sought to evaluate whether those who had surgery on their dominant hand had a functional outcome that differed from non-dominant surgery patients at medium term follow up. The null hypothesis of this study being that there is no difference between the functional outcome of dominant and non-dominant hand rotator cuff repair.

## 2 METHODS

We conducted a retrospective review of all rotator cuff repairs carried out in a single surgeon practice over the course of a single calendar year. All rotator cuff repairs were carried out by the senior author, a fellowship trained shoulder surgeon.

Patients were assessed pre operatively by the lead surgeon and scheduled for surgery on the basis of clinical and radiological assessment. Only patients with repairable full thickness rotator cuff tears of the supraspinatous and postero-superior cuff on MRI were included. Patients with partial thickness tears, irreparable tears or subscapularis tendon involvement were excluded. Additionally those with labral pathology requiring repair, arthritic change of the glenohumerual or acromioclavicular joints or previous surgery on the same shoulder were excluded.

Arthroscopic single row rotator cuff repair was performed for all cases. Diagnostic arthroscopy was performed with an identical 30 degree scope. Tear location, configuration and level of retraction were documented in the operative note. Retraction was graded sequentially as not retracted, retracted to humeral head or retracted to the glenoid. Tear edges were debrided and greater tuberosity was decorticated. Repair was performed using tendon to bone suture anchors. For larger or retracted tears side to side repair supplemented suture anchor repair.

Post operatively patients followed an identical rehabilitation protocol consisting of immobilistaion in a sling for 3 weeks followed by a graded physiotherapy rehabilitation programme. This comprised of an initial phase of range of motion exercises for 4 weeks followed by a muscle-strengthening regime.

All patients identified by our review of theatre records from 2013 were scheduled for 3 year follow up. Demographic data was recorded including gender, date of birth, operative side, date of initial assessment and date of surgery. To carry out our follow up we gained informed consent for inclusion in the study and calculated functional outcome and satisfaction scores using the *L’insalata* shoulder score questionnaire [15]. This comprises of individual scores for pain, daily activities, recreational and work activities, overall satisfaction as well as a combined overall score. Outcomes were score from 0-10 with 0 being the worst score and 10 being the best. Hand dominance was recorded for all patients as part of the questionnaire as well as date of questionnaire completion.

Details of the study, the questionnaire with brief instructions and a consent form were posted to patients. A stamped addressed envelope was included for reply. In order to achieve as high a level of response as possible those who did not respond by post were contacted by telephone on a minimum of 2 occasions.

Functional scores for all patients were collected and entered into a password-protected database. Statistical comparison of functional scores between patients’ surgically operated dominant limb and surgically operated non-dominant limb was performed using a multivariate linear regression analysis. Independent variables included in our analysis were age, gender, tear location, level of tear retraction, time from initial assessment to surgery and time from surgery to follow up. Analysis was performed with SPSS version 22 [16]. For all statistical tests a value of *P* < 0.05 inferred statistical significance.

## 3 RESULTS

178 patients were identified that met our inclusion criteria. Of those 144 responded, a response rate of 81%. Of those who completed follow up 122 patients (84.7%) were right handed and 22 patients (15.3%) were left handed. Overall 92 patients (63.9%) had surgery on their dominant side, whilst 52 patients (36.1%) had surgery on their non-dominant side. No participant had bilateral rotator cuff repair within the study period.

There was a mean age of 63+/- 10.1 years in the dominant group and 62 +/- 8.6 years in the non dominant group. There were 48 females and 44 males in the dominant group, with 27 females and 25 males in the non-dominant group. Hand dominance was significantly associated with side of rotator cuff tear (P=0.005).

Univariate analysis found no effect of age, gender, tear location, retraction, assessment to surgery time or assessment to follow up time on outcome. Gender did have a significant effect on outcome score (p = 0.03).

The mean overall outcome score was marginally higher in the dominant surgery group with a mean of 89.8 +/- 14.2 compared with a mean of 87.4 +/- 17.5 in the non-dominant group. Multi-variate analysis including age, gender, tear location, tear retraction, assessment to surgery time and surgery to follow up time as individual input variables revealed this difference to be non significant (p = 0.4).

Analysis of the individual component scores of the *L’insalata* questionnaire showed a trend towards better scores in the dominant side surgery group. This is depicted in the table below (Table **1**). Those in the dominant sided surgery group had greater mean scores in the global assessment, pain, daily activities, recreations and satisfaction components, however none of these differences reached statistical significance.

## 4 DISCUSSION

We found no difference in patient reported outcome measures between dominant and non-dominant hand rotator cuff repair at 3 year follow up.

Our dominant hand is at more risk of rotator cuff tear [10]. Given the additional demands in daily life on patients dominant hand we sought to investigate if the patient reported outcome following surgery differed between dominant and non-dominant side. Our findings are helpful in terms of counseling patients pre operatively who may worry about return to function or the morbidity associated with operating on their dominant hand. A trend towards marginally superior outcomes in four of the five individual component scores for dominant hand surgery adds further reassurance.

A study by Woolard et al found greater improvement in patient reported disability following surgery on the dominant hand [17]. This study measured post-operative outcome at 6 months whereas our study showed longer term follow up at 3 years. It is therefore a clinically important finding that the functional outcome at 3 years does not differ and offers useful evidence for counseling patients’ pre operatively.

Patient reported outcome measures are an important research tool for evaluating healthcare outcomes, however they are dependent on numerous factors. Studies evaluating shoulder pain outcome scores in a primarily degenerative rotator cuff tear population found that psychosocial factors such as anxiety, catastophising and depression as well as social factors such as education, language and professional qualification were associated with poorer outcome scores and less perceived improvement in function [18, 19]. Various factors have been shown to impact upon the outcome of rotator cuff repair surgery as discussed in the introduction. Factors such as age, gender and time for tear to surgery are more consistently cited as having an affect [11]. Our study did not find an impact on outcome from a number of factors including age, time to surgery from assessment and time to follow up. However, we did find that outcome score varied with gender. No conclusion can be drawn from this finding however without analysis of baseline values.

Moosmayer et al showed improved outcomes for 1 year and 5 year follow up for operative treatment over non operative treatment [20, 21]. The difference is small at 5 years however and may not be clinically significant [21]. Other studies have shown one year outcome scores to be similar comparing operative and non operative treatment in randomised control trials [22, 23]. However, at 3 years pain and activities of daily living scores were better in the operative group compared to conservative treatment [9]. In the context of our study this is relevant in that medium term outcomes are equivocal between dominant and non-dominant sides.

We found that patients were more likely to present for rotator cuff repair on their dominant hand, a finding which has been shown in systematic review [10]. Whether this is due to increased incidence of rotator cuff pathology in the dominant arm of the population due to mechanical factors or whether patients are more likely to seek operative management if affected on their dominant side is not clear. Potential for further research exists to clarify if this increased rate of surgical intervention on the dominant side is due to a greater negative impact on quality of life and activities of living.

The strengths of our study lie in the homogeneity of the population treated by a single surgeon with extensive expertise in one calendar year. Follow up outside of the initial immobilisation and recovery period negates the effect of initially reduced ability to perform activities with their dominant hand. We also included extensive co-variate analysis in our statistical tests. Our study is limited however by not having baseline or early post-operative functional outcomes. Similar to the study by Woollard [17] we could have compared change in outcome or included baseline values as a co-factor in our analyses. Verification of repair integrity as well as further reducing the attrition rate would strengthen our analysis.

## CONCLUSION

No difference was found in the functional outcome of rotator cuff repair between dominant and non-dominant side surgery. This information will help in counselling patients who are concerned about the potential impact of rotator cuff repair on the function of their dominant hand.

## Figures and Tables

**Table 1:** Functional outcome scores for participants who had surgery on their dominant or non-dominant limb. Scale of 0-10 with score of zero depicting very poor outcome and 10 indicating excellent outcome.

		**Global Assessment**	**Pain**	**Daily Activities**	**Recreation**	**Work**	**Satisfaction**
**Dominant limb** **(n=92)**	**Mean +/- STDEV**	8.4 ± 2.2	9.0 ± 1.6	9.1 ±1.3	8.8±1.7	9.3±1.4	8.3±2.2
**Non-Dominant limb** **(n= 52)**	**Mean +/- STDEV**	8.1 ± 2.7	8.9±1.9	8.9± 1.5	8.6±1.9	9.3 ± 1.6	7.8 ± 2.7
		P=0.63	P=0.695	P=0.352	P=0.685	P=0.93	P=0.335
